# Optimizing predictions of environmental variables and species distributions on tidal flats by combining Sentinel-2 images and their deep-learning features with OBIA

**DOI:** 10.1080/01431161.2024.2423909

**Published:** 2024-11-19

**Authors:** Logambal Madhuanand, Catharina. J. M. Philippart, Wiebe Nijland, Steven M. de Jong, Allert I. Bijleveld, Elisabeth A. Addink

**Affiliations:** a Department of Physical Geography, Utrecht University, Utrecht, The Netherlands; b NIOZ Royal Netherlands Institute for Sea Research, Department of Coastal Systems, Texel, The Netherlands

**Keywords:** Sediment characteristics, macrozoobenthos, OBIA, Sentinel-2, autoencoder, random forest

## Abstract

Tidal flat ecosystems, are under steady decline due to anthropogenic pressures including sea level rise and climate change. Monitoring and managing these coastal systems requires accurate and up-to-date mapping. Sediment characteristics and macrozoobenthos are major indicators of the environmental status of tidal flats. Field monitoring of these indicators is often restricted by low accessibility and high costs. Despite limitations in spectral contrast, integrating remote sensing with deep learning proved efficient for deriving macrozoobenthos and sediment properties. In this study, we combined deep-learning features derived from Sentinel-2 images and Object-Based Image Analysis (OBIA) to explicitly include spatial aspects in the prediction of tsediment and macrozoobenthos properties of tidal flats , as well as the distribution of four benthic species. The deep-learning features extracted from a convolutional autoencoder model were analysed with OBIA to include spatial, textural, and contextual information. Object sets of varying sizes and shapes based on the spectral bands and/or the deep-learning features, served as the spatial units. These object sets and the field-collected points were used to train the Random Forest prediction model. Predictions were made for the tidal basins Pinkegat and Zoutkamperlaag in the Dutch Wadden Sea for 2018 to 2020. The overall prediction scores of the environmental variables ranged between 0.31 and 0.54. The species-distribution prediction model achieved accuracies ranging from 0.54 to 0.68 for the four benthic species). There was an average improvement of 21% points on predictions using objects with deep learning features compared to the pixel-based predictions with just the spectral bands. The mean spatial unit that captured the patterns best ranged between 0.3 ha and 13 ha for the different variables. Overall, using both OBIA and deep-learning features consistently improved the predictions, making it a valuable combination for monitoring these important environmental variables of coastal regions.

## 1. Introduction

Tidal flat ecosystems are coastal wetlands of high ecological importance occurring between land and sea (Reise 1985; Wolff 2013) that get alternatively submerged and exposed by the tides. This ecosystem provides an important habitat for many animals such as migratory birds and juvenile fish and offers essential ecosystem services for humans such as protection against storms, and commercial grounds for fishing (Kirwan and Megonigal 2013). Among the many factors indicating the health of an ecosystem, the presence and distribution of macrozoobenthos are of high relevance as they are mostly resident and provide food for higher trophic levels such as birds and fish (Drent et al. 2017; Herman et al. 1999). The species distribution of macrozoobenthos is strongly correlated to the sediment characteristics (Folmer et al. 2017; Beukema 1976; Wal et al. 2008), which in turn are influenced by both hydrodynamic and morphological factors (Andersen and Pejrup 2001; Kuipers, de Wilde, and Creutzberg 1981; Reise 1985).

Globally, the quantity and quality of tidal flat ecosystems are in decline due to sea level rise, coastal development activities, and other anthropogenic impacts (Murray et al. 2019). Accurate and up-to-date information is essential for the management and protection of the tidal ecosystem conditions (Mao et al. 2020). Monitoring benthic and sediment properties is challenging, however, due to low field accessibility, limited exposure time, and high ecological vulnerability. Along with these challenges, the dynamic nature of these environmental variables necessitates repeated and representative sampling at a high resolution in both time and space.

Remote sensing provides a cost-effective data collection procedure to capture spatial patterns for extensive and inaccessible areas. This also holds for tidal flat systems, even though these provide additional challenges such as limited exposure time (Barale and Gade 2018) and low spectral contrast (Madhuanand et al. 2023). Macrozoobenthos lives at the surface or on the subsurface and is, therefore, often not directly visible. Their habitat is defined by environmental factors such as exposure time, sediment composition (median grain size, mud content), and abundance of microphytobenthos (Compton et al. 2013). These factors do affect surface characteristics and their spatial arrangement, hence (partly) reflect the distribution of macrozoobenthos. Analysing the surface patterns is therefore an indirect approach to studying macrozoobenthos. To enhance the information extraction from satellite images, Madhuanand et al. (2023) developed an autoencoder deep-learning model. These use filters to create features in the encoding phase by capturing contextual and multispectral information. The models aim to reconstruct the input image, so the created features optimally capture spectral and spatial information of the original image. These features were extracted and combined with spectral bands to predict tidal-flat properties. Accuracy values increased by 15% points, showing that the satellite images contain valuable information on tidal flats despite the low spectral contrast. Through unsupervised learning, autoencoders also leverage a larger volume of imagery for feature extraction without requiring large amounts of field-collected data.

An aspect that was not studied by Madhuanand et al. (2023) is the potential further improvement of this accuracy by optimizing the spatial observation unit. The filters in the deep learning model operate in the spectral and/or spatial domain (Simonyan, Vedaldi, and Zisserman 2014). However, it is important to note that the fundamental spatial unit corresponds to an individual pixel. Ecosystems on tidal flats are strongly affected by physical environmental processes with clear spatial autocorrelation, that varies across scales (Lyashevska, Brus, and van der Meer 2016). When the spatial observation unit matches elements of the ecosystem studied, this could lead to further improvements in the prediction of its properties (Addink, De Jong, and Pebesma 2007). Within the field of remote sensing, object-based image analysis (OBIA) (Blaschke 2010; Hay and Castilla 2008) offers the opportunity to look for optimal spatial units. OBIA creates objects by grouping neighbouring pixels and uses those as the spatial entity. While pixel-based image analysis focuses on spectral properties, OBIA allows the synchronous use of spectral, geometric, and contextual information (Blaschke 2010). It is hence a sensible approach to ecological and environmental studies (Blaschke et al. 2014; Hay and Castilla 2008).

**Figure 1.**
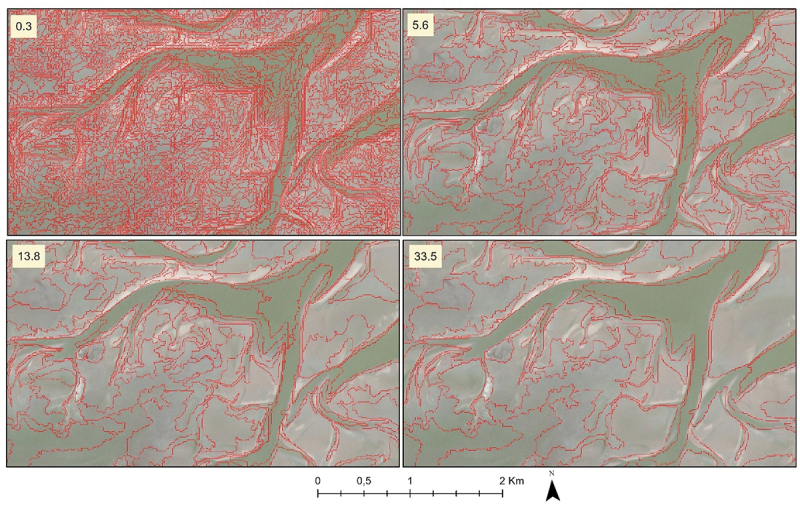
Examples of segmented images created with different object heterogeneity (mean area in ha indicated in the upper left corner) of a tidal flat in the Dutch Wadden Sea overlayed on Sentinel-2 image, 2019.

OBIA segments images into objects (Figure 1) by merging groups of pixels with similar characteristics using a bottom-up approach (Addink, Van Coillie, and de Jong 2012; Baatz and Arno 2000; Burnett and Blaschke 2003; Hay and Castilla 2008). Because the objects are created from the input data, they are considered to segment the image into consistent spatial units that follow the spatial structure of the area. When going from higher to lower spectral similarity, the object size will increase. OBIA showed improvements in various ecological studies with the contextual and spatial information obtained through neighbouring pixels rather than by just using the per-pixel information (Addink, De Jong, and Pebesma 2007; Ma et al. 2017; Nijland et al. 2009). Addink, De Jong, and Pebesma (2007) used OBIA for predicting vegetation parameters like above-ground biomass and leaf area index to identify optimal observation units. In recent years, several studies used OBIA techniques in coastal ecosystems either for classification tasks such as habitat mapping of corals or seagrass (Monteiro et al. 2021; Phinn, Roelfsema, and Mumby 2012; Roelfsema et al. 2018; Tian et al. 2008). This hybrid method, which combines deep learning features with OBIA, ensures the integration of state-of-the-art techniques while incorporating valuable spatial context.

In this study, we aimed to evaluate the use of OBIA methods to predict macrozoobenthos and sediment characteristics of tidal flats from Sentinel-2 images and their deep-learning features. We considered the following research questions:

1. How does the prediction accuracy change if OBIA is included in the prediction of three types of environmental variables: sediment (median grain size, silt content), macrozoobenthos (total biomass, species richness) and species distribution (*Arenicola marina, Macoma balthica, Oligochaeta, Urothoe sp*.)?
2. What are the parameters to create the optimal spatial units to predict these environmental variables?

To answer these questions, we used Sentinel-2 images and the deep-learning features extracted from a variational autoencoder model (VAE) trained on data from the Dutch Wadden Sea (Madhuanand et al. 2023) to define object sets. We used these object sets to predict the following variables: median grain size, silt content, macrozoobenthic biomass, and species richness of the tidal flats in the Dutch Wadden Sea using a random forest regressor. The optimal definition of objects and the corresponding spatial units for all variables were determined based on the performance of the prediction model. We then compared the OBIA model predictions to pixel-based predictions to determine whether the prediction accuracy increases and assess the added value of OBIA for mapping and monitoring environmental variables of coastal ecosystems.

## 2. Materials & methods

### 2.1. Study area

**Figure 2.**
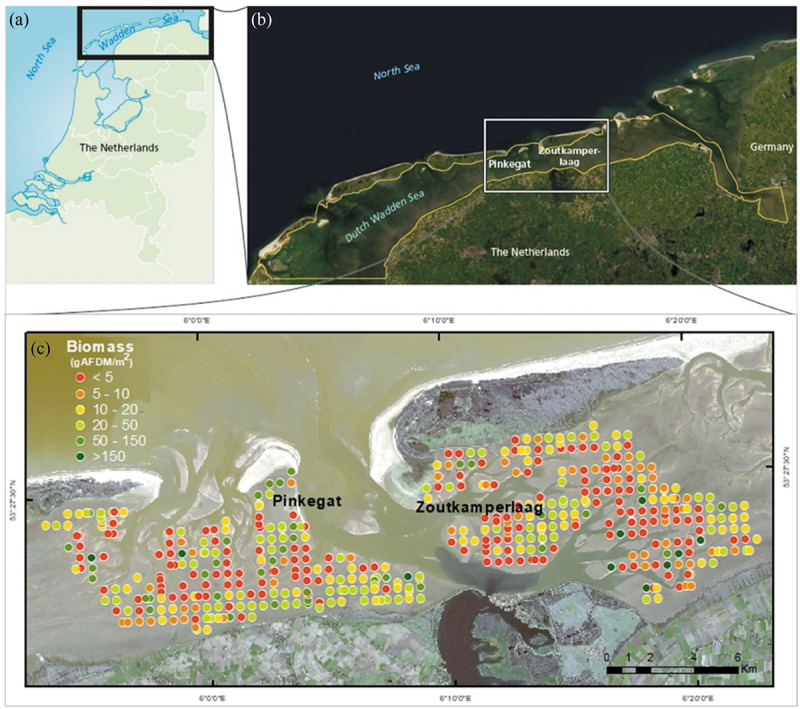
(a) and (b) Location of the study site, (c) total biomass observations (gAFDM m^−2^) in Pinkegat & Zoutkamperlaag, Dutch Wadden Sea for 2019 (Bijleveld et al. 2012) overlayed on a Sentinel-2 image (2019-02-15).

The study site consists of two tidal basins in the Dutch Wadden Sea, Pinkegat (53°25N, 5°46E) and Zoutkamperlaag (53°26N, 6°15E), which jointly cover an area of 320 km^2^ (Figure 2). The Wadden Sea extends from the Netherlands to Denmark, with the Dutch Wadden system covering an area of 2500 km^2^. Due to its universally outstanding value, this area was designated as a UNESCO Natural World Heritage in 2009. The ecosystem is composed of intertidal flats, drainage gullies, deeper channels, and shallower subtidal flats.

The Tidal Basin of Zoutkamperlaag covers an approximate area of 200 km^2^, whereas Pinkegat encompasses about 120 km^2^. The tidal range is 2 m in both basins and the tidal wave propagates from west to east along the coast. The median grain size follows a general trend with finer particles close to the mainland shores and coarser sand near tidal inlets and the barrier islands (Figure 2). Conversely, the percentage of silt content is generally higher close to the mainland shores and lower near tidal inlets and these barrier islands. The average median grain size in the region varies between 140 and 145 µm and silt content varies between 13 and 15% (Bijleveld et al. 2012). Macrozoobenthos biomass and richness are higher in the regions closer to the mainland coast and further away from the channels while they tend to be lower near the tidal inlets located between the islands. The average species richness is 7 to 9 species and the biomass ranges between 25 and 30 g Ash Free Dry Mass (AFDM)/m^2^ (Bijleveld et al. 2012).

### 2.2. Data

#### 2.2.1. Image data

Sentinel-2 (L1C) images (User Guides 2015) acquired between 2018 and 2020 that were both cloud-free and captured during low tide conditions, were downloaded from the USGS Earth Explorer. Sentinel-2 MSI contains 13 spectral bands varying from visible to shortwave infrared with a spatial resolution ranging from 10 m to 60 m. The four bands of 10 m resolution, Blue (490 nm), Green (560 nm), Red (665 nm), and Near-Infrared (NIR, 842 nm) were used in this study. The two tidal basins were covered with two Sentinel-2 tiles (each 100 × 100 km^2^) and the respective tide levels above the lowest astronomical tidal (LAT) levels during image acquisition were recorded from the nearest tidal stations, specifically Schiermonnikoog for the Zoutkamperlaag basin and Holwerd for the Pinkegat basin (Rijkswaterstaat n.d.). (Table 1).

**Table 1.** Selected sentinel-2 images for the Pinkegat and Zoutkamperlaag basins, along with corresponding sea levels (above LAT) at the time of image acquisition (2018–2020) (Rijkswaterstaat, n.d.).

Date of image acquisition	Tidal height above LAT (in m)	Time to low tide (in hh:mm)
*Pinkegat*		
17 November 2018	1.24	+00:42
15 February 2019	0.92	00:00
5 April 2020	1.72	−02:36
*Zoutkamperlaag*		
6 August 2018	0.89	+1:12
26 August 2019	0.74	00:00
5 April 2020	1.27	−02:06

Madhuanand et al. (2023) trained a variational autoencoder model using 17 Sentinel-2 images (2018–2020) covering the entire Dutch Wadden Sea tidal flats. The four band Sentinel-2 image patches of the tidal flats were given as input to the model that learned to reconstruct the tidal flats. From the first encoded layer of the trained model, 64 features were extracted. These 64 deep-learning features were then included in the prediction of environmental variables and showed to enhance the information extraction from the images of the tidal flats. In this study, we used those 64 deep-learning features obtained from each of the six Sentinel-2 images as input data (Table 1).

#### 2.2.2. Field data

The study site has been monitored since 2008 by the Royal Netherlands Institute for Sea Research (NIOZ) in the framework of the annual campaign ‘Synoptic Intertidal BEnthic Survey’ (SIBES). The SIBES network has ~4800 observation points (10% random points) sampled 500 × 500 m on the tidal flats of the entire Dutch Wadden Sea (Bijleveld et al. 2012; Compton et al. 2013) sampled at. At each sample location, two cores of a combined area of 0.0173 m^2^ are taken to a depth of ~25 cm to collect macrozoobenthos. Besides, a centrifuge tube is used to collect sediment samples to a depth of ~4 cm. From the extensive list of variables, the following were included in the predictions for this study:

- Median grain size (µm)
- Silt content (Volume % < 63 µm)
- Total biomass per sampling station (gAFDM m^−2^)
- Species richness: Number of unique species per sample
- Presence/absence of species

In addition, one species (or species group) was selected from each of the four common taxonomy classes (Mollusca, Oligochaeta, Crustaceans, Polychaetes) which was present in 40–60% of the SIBES points (Table 2). This occurrence rate of approximately 50% reduced the effect of the imbalance between presence/absence in the predictions later on. The final dataset included location, median grain size, silt content, biomass, species richness, and presence/absence of the four species (groups) for all point observations in the two tidal basins. For the variables silt content and biomass, a logarithmic transformation was applied due to their skewed data distribution. Around 200 to 250 point observations are available per tidal basin per year, adding up to a total of 1400 points from 2018 to 2020 (Table 3).

**Table 2.** Selected species for the distribution maps with the number of present/absent observations for the 3 years.

Taxonomy class		Points present		Points absent		
	Species name	Pinkegat	Zoutkamper laag	Pinkegat	Zoutkamper laag	Presence %
Polychaetes-I	*Arenicola marina*	296	265	373	454	41
Mollusca	*Macoma balthica*	356	339	313	380	52
Oligochaeta	*Oligochaeta*	229	341	440	378	42
Crustaceans	*Urothoe sp*.	289	218	380	501	37

**Table 3.** Descriptive statistics of the four environmental variables.

		Median grain size (µm)			Silt content (%)			Biomass (gAFDM/m^2^)			Species richness (no. of species/sample)			
Region	Field sampling dates	Mean	Std	Min-Max	Mean	Std	Min-Max	Mean	Std	Min-Max	Mean	Std	Min-Max	No. of points
*Pinkegat* *+* *Zoutkamperlaag*	1 August 2018 – 7 August 2018	145.2	35.1	32.4-231	14.3	13.6	1.5-66.9	32.1	48.9	0.02-482.7	8.8	3.82	1-21	455
	7 August 2019 – 31 August 2019	141.8	36.7	21-222	15.85	14.7	1.25-78.1	23.54	48.6	0.005-684.1	7.65	3.15	1-18	462
	21 June 2020 – 24 June 2020	146.9	34.6	20.6-222.6	13.91	13.36	1.55-82	21.2	43.7	0.02-546.5	8.5	3.68	1-19	471

**Figure 3.**
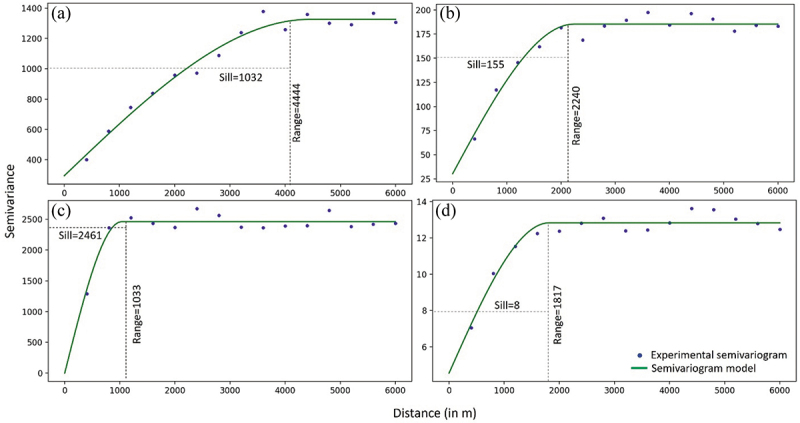
Semi-variograms of the four environmental variables for 3 years and two regions combined. (a) median grain size, (b) silt content, (c) biomass, and (d) species richness.

2.2.2.1 Semivariogram: The spatial dependence between the field observation points was assessed using semivariograms (Curran 1988). A semivariogram considers pairwise distance and the variance between the points. A spherical model was fitted for the four environmental variables (Figure 3) and the four species distributions (Figure A1). Each model was characterized by the parameters sill and range. The total variability in observation and the distance after which the observations are independent were explained by sill and range.

The range values of the four environmental variables varied from 4444 m to 1033 m for the four variables. Each variable exhibited a distinct spatial dependence pattern, with those having shorter ranges having less variance and stronger spatial correlation within a shorter distance. Among these variables, median grain size displayed the largest range size, showing that its spatial dependence extended over a greater distance compared to the other three variables. Similarly, for the species distributions (Figure A1), the variograms varied from 2476 m to 1226 m, with Oligochaeta species group exhibiting a larger range size compared to the individual species. Overall, the parameters of the spherical model provided valuable insights into the spatial relationships and variability of both environmental variables and species distribution in the study area.

### 2.3. Approach

#### 2.3.1. OBIA segmentation

We used Multi-Resolution Segmentation (MRS) to segment the image into spatial units based on their spectral, spatial, and contextual characteristics. MRS is a bottom-up approach that starts by merging randomly selected pixels with adjacent pixels until the heterogeneity within the new object is too large (Baatz and Arno 2000; Benz et al. 2004). The objects are created with different input combinations based on scale, shape, and compactness. The scale parameter determines the heterogeneity of the features or objects that the segmentation algorithm identifies and has a strong impact on the final size of the objects. The shape parameter influences the regularity or irregularity of the borders of the created objects. It helps define the desired shape characteristics of the objects in the image. The compactness parameter controls how tightly the pixels within a segment are grouped together. We selected the ranges for Scale (10 to 200 with an increment of 10), Shape (0.1 to 0.9 with an increment of 0.4), and Compactness (0.1 to 0.9 with an increment of 0.4) after a short experiment. Object sets with different spatial units were created for these combinations, from which we selected the optimal combination for each environmental variable based on the prediction accuracies.

Object properties are divided in three categories; spectral, textural, and geometrical (Blaschke 2010). Here, the objects were defined using just the spectral bands or the spectral bands in combination with the deep learning features. Spectral properties of the objects were described by the mean and standard deviation of the pixel values from the input layers. Textural properties of the objects were described using Gray Level Co-occurrence Matrix (GLCM) variables (Haralick, Sam Shanmugam, and Dinstein 1973). GLCM calculates the frequency of pairs of pixels with specific values and in a specified spatial relationship that occurs in an image. We used five GLCM variables (contrast, correlation, entropy, homogeneity, dissimilarity) that describe spatial pattern within objects. The geometry of the objects was described using area, compactness, and density which capture the structural information from the input images.

**Figure 4.**
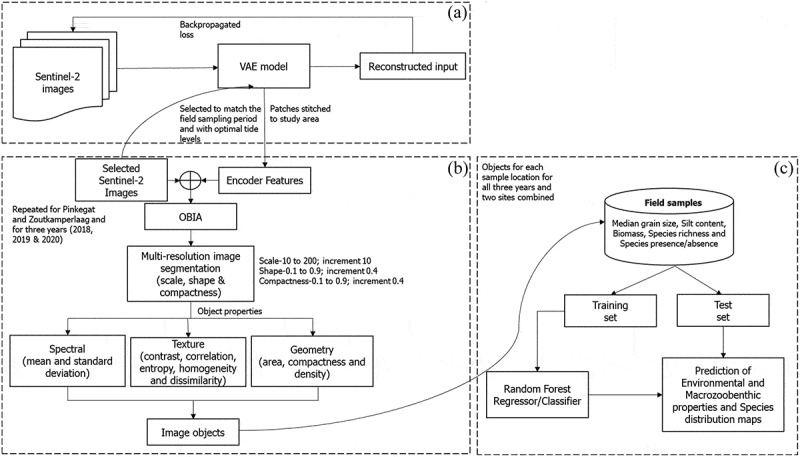
Workflow for predicting the environmental variables using object-based image analysis (a) deep learning features extraction, (b) object creation, (c) prediction of the variables.

The three-step approach (Figure 4) starts with extracting deep learning features from the Sentinel-2 images followed by segmentation (OBIA) and variable extraction, followed by the prediction and validation process. OBIA was implemented using the software package of eCognition (Trimble n.d.). The objects were defined using either the Sentinel-2 four spectral band images (Table 1) or with the 64 deep-learning features or a combination of both spectral bands and features for the Pinkegat and Zoutkamperlaag tiles. In this study, only the tidal flat regions were used for object creation, while the land and sea regions were excluded. The segmentation process used scale parameters ranging from 10 to 200, with an increment of 10, thus creating 20 different object definitions for each input combination. A larger scale parameter resulted in a higher number of objects with less homogeneity. Both shape and compactness parameters ranged from 0.1 to 0.9, with an increment of 0.4. It was observed that shape and compactness values greater than 0.1 which lead to a smoother object border and pixel closeness, did not lead to noticeable changes in accuracy. Therefore, we continued our work using shape and compactness values set at 0.1.

Three sets of input combinations were used for object definition: spectral bands only, features only, spectral bands, and features together. Three categories were used to describe the defined objects: spectral, texture, and geometry. The combination of object definition and object properties led to a set of five object entities (Table 4(a-e)). Object sets a) and b) include objects that are defined by spectral bands only. Objects are characterized by mean and standard deviations across four spectral bands (sets a and b) plus five textural properties for each band along with three geometrical properties (set b). Object set c) was defined using just the 64 deep learning features and the object properties include the mean and standard deviation of these features. Object sets d) and e) were defined by the four spectral bands and 64 deep-learning feature layers, i.e. 68 layers. Object properties include mean and standard deviation of each layer (sets d and e) plus five textural properties along with the three geometrical properties (set e). For all input combinations, the objects were defined over the selected range of scale, with shape and compactness set to 0.1.

**Table 4.** Variables used in segmentation and the number of derived properties used in prediction (S = spectral bands, D = Deep-learning features).

		Spectral bands (SP)	Deep-learning features (FEA)	Texture & Geometry per band (TXT,GEO)	
	Segmentation variables	Mean and Standard Deviation	Mean and Standard Deviation	Contrast, Correlation, Entropy, Homogeneity, Dissimilarity, Area, Compactness, Density	Total number of object properties
a)	4S (SP)	4+4	–	–	8
b)	4S (SP+TXT+ GEO)	4+4	–	5*4S+3	31
c)	64D (FEA)	–	64+64	–	128
d)	4S+64D (FEA + SP)	4+4	64+64	–	136
e)	4S+64D (FEA +SP + TXT + GEO)	4+4	64+64	5*4S + 5*64D+3	479

### 2.4. Prediction model

For each of the five object sets (Table 4) with unique realizations for each of the 20 scale parameters, random forest models (Breiman 2001) were developed and trained along with the sediment, macrozoobenthic, and distribution indicators. The ability to handle non-linearity from the data and the reliability of fitting the model with various input variables made random forest preferable for our task. For each field observation, the properties of the corresponding object (location and year) were extracted for the 20 realizations within the five object sets. If an object corresponded to multiple field points, each point was treated as an independent entity associated with the same object segment.

The extracted objects for each object set of the environmental variables were divided into 90% training and 10% testing data points. Models were trained using the combined data from both study sites for the 3 years, and were used to predict four variables as well as the presence/absence of the entire set of objects. Due to the limited number of field data, a K-fold cross-validation (Jung and Hu 2015) strategy was chosen to reduce overfitting and to determine a stable estimation of the accuracy of the model. We applied a 10-fold cross validation with three repetitions averaged to estimate the model accuracy. Several experiments were run to set the hyperparameters, including the number of trees, minimum number of data points at each node and the maximum number of features considered for split. The predictions of the ecological variables using the regressor were evaluated using R^2^ (coefficient of determination) value. Positive R^2^ values indicate that the model performs better than taking the mean value of the input data. The quality of the distribution maps was evaluated using the F1 score which is a harmonic mean of precision and recall (Chinchor 1992). Perfect precision and recall will result in the highest F1 score of 1.0 or a lowest score of 0.

## 3. Results

### 3.1. Optimal object sets and scale values for the environmental variables

**Figure 5.**
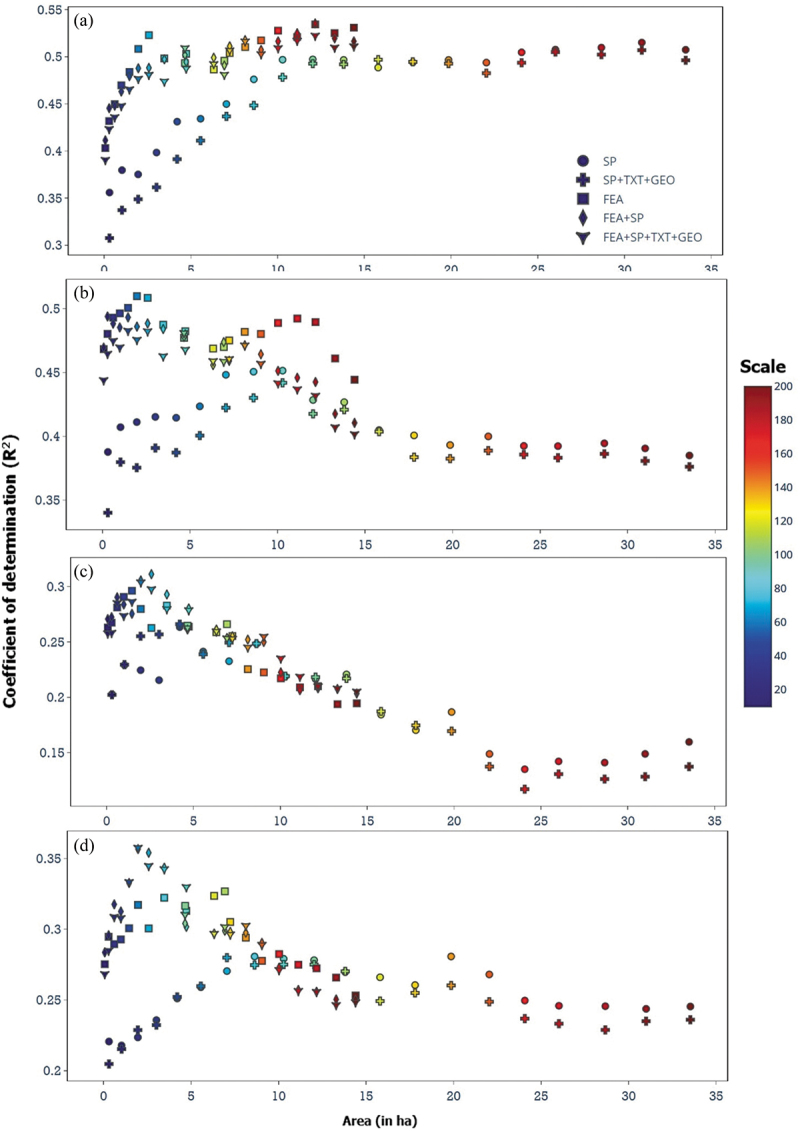
Coefficient of determination (R^2^) for the four variables with different object size (in ha). Colour indicates scale 10–200 (colour bar) and the symbols represent the five object sets (Table 4). (a) median grain size, (b) silt content, (c) biomass, and (d) species richness.

Each of the four ecological variables and the spatial distribution of each of the four species was predicted for the five object sets (Table 4) at 20 different scale values (Tables A3 and A4). Best results were obtained for sets c), d), and e) which included deep learning features in the object definition with scale values ranging from 60 to 180 (Figure 5).

The range of prediction scores for the sediment properties was larger than that of biomass and species richness. The prediction scores for biomass and species richness were clustered together over different object sizes and object sets (Figure 5). Median grain size produced the best prediction score of 0.54 for object set d) (spectral bands and deep-learning features) at object size 12 ha (scale 180) (Figure A2). For silt content, a prediction score of 0.51 was achieved with object set c) (deep-learning features) with an object size of 2 ha (scale 60). For biomass and species richness, the bpredictionions scores were 0.31 and 0.36 for object set d) (spectral bands and deep-learning features) at object size 2.6 ha and 2 ha (scale 70 and 60).

The five object sets were also used to predict presence/absence distributions for four selected species/species groups. Similar to the four environmental variables, object sets that included the deep learning features provided the highest F1 score for the presence/absence predictions for each species (Table A4). For the polychaeta, *Arenicola marina* the F1 prediction score of 0.54 was achieved for object set e) (spectral bands, features, textural, and geometry properties) at object size 13 ha (scale 190). The mollusc *Macoma balthica* was predicted with an F1 score of 0.68 with object set d) (spectral bands, and deep-learning features) at object size 0.3 ha (scale 20). For the group of *Oligochaeta*, the predictions were 0.64 for the object set e) (spectral bands, features, textural, and geometry properties) at object size 3.5 ha (scale 80). For the group of crustaceans *Urothoe sp*., the predictions were 0.57 for object set c) (deep-learning features) at object size 11 ha (scale 170). The species *Macoma balthica* produced the best score for lower object size, while the other species (*Arenicola marina*) and species groups (*Oligochaeta* and *Urothoe sp*.) needed a higher range.

The spectral bands along with deep-learning features consistently yielded results that were similar to the optimal object set (printed in bold) for each variable and species (Table 5). This suggests that the combination of spectral bands and deep-learning features showed the potential to effectively replace other object sets in our analysis. So we choose for all four variables & species distributions, the best object set as spectral bands with deep-learning features for the selected object size.

**Table 5.** Best predictions per object set for the four environmental variables and the four species. The object size relates to the scale value with the best overall prediction (printed in bold).

	a) Spectral bands (SP)	b) Spectral bands + texture + geometry (SP + TXT + GEO)	c) Deep-learning features (FEA)	d) Spectral bands + deep- learning features (FEA + SP)	e) Spectral bands + deep-learning features + texture + geometry (FEA + SP + TXT + GEO)	Object size (in ha)
**Variables (R**^**2**^**score)**						
Median grain size (µm)	0.509	0.502	0.534	**0.535**	0.522	12.2
Silt content (%)	0.423	0.401	**0.509**	0.496	0.476	2.0
Biomass (gAFDM/m^2^)	0.233	0.249	0.263	**0.311**	0.298	2.6
Species richness (no. of species/sample)	0.259	0.259	0.317	**0.357**	0.347	2.0
**Species (F1 score)**						
*Arenicola marina*	0.504	0.511	0.509	0.529	**0.539**	13.2
*Macoma balthica*	0.663	0.627	0.673	**0.682**	0.664	0.3
*Oligochaeta*	0.559	0.577	0.617	0.633	**0.642**	3.5
*Urothoe sp.*	0.524	0.525	**0.566**	0.559	0.539	11.1

**Figure 6.**
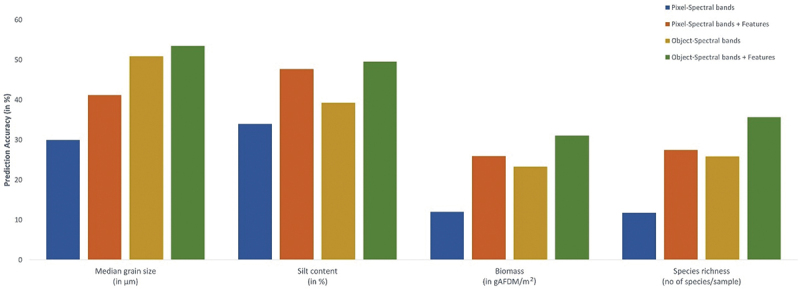
Prediction accuracies compared between pixel- and object-based analysis for the spectral bands and deep-learning features for the four variables.

The prediction scores of all four variables obtained using the object sets were compared to those of the pixel-based prediction model of Madhuanand et al. (2023) (Table A5). We compared the two object sets, spectral bands a) and spectral bands with deep learning features d) of the object-based model to that of the pixel-based model which used spectral bands and spectral bands with deep learning features for pixel predictions. For the four variables, the object-based model using spectral bands along with deep-learning features showed an improvement of 8.9% points on average than the model which uses just the spectral bands. Overall, the object-based approach with deep-learning features improved the predictions by 21% points on average for all variables considered compared to a pixel-based approach with just the spectral bands (Figure 6). Also, the R^2^ improved by 13% points on average when using the object-based approach rather than the pixel-based one with just spectral bands. With spectral bands and deep learning features, the R^2^ improved by 7% points on average when using the object-based approach than the pixel based. The object-based model showed a consistent improvement in prediction performance for all the variables compared to pixel-based approach with/without features.

### 3.2. Predicted spatial patterns and descriptive analysis

**Figure 7.**
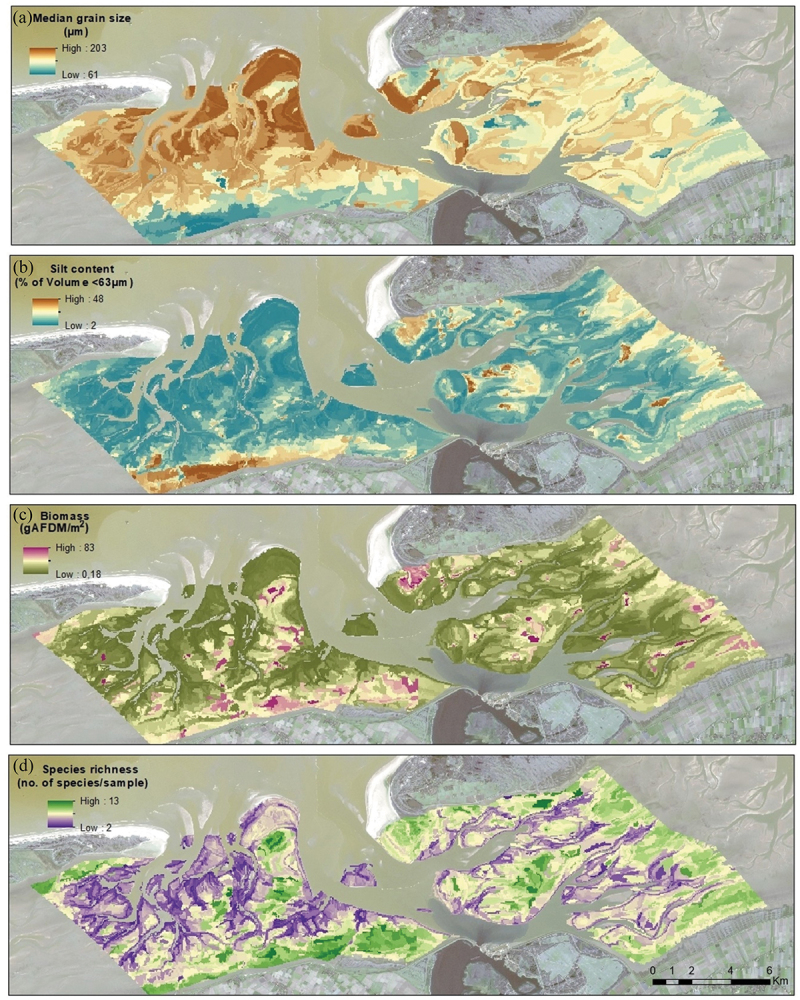
Predicted maps of the four variables (with optimal object size) for 2019 using object set d) (spectral bands and deep-learning features (FEA+ SP)). (a) median grain size (12 ha), (b) silt content (2 ha), (c) biomass (2.6 ha), (d) species richness (2 ha).

**Figure 8.**
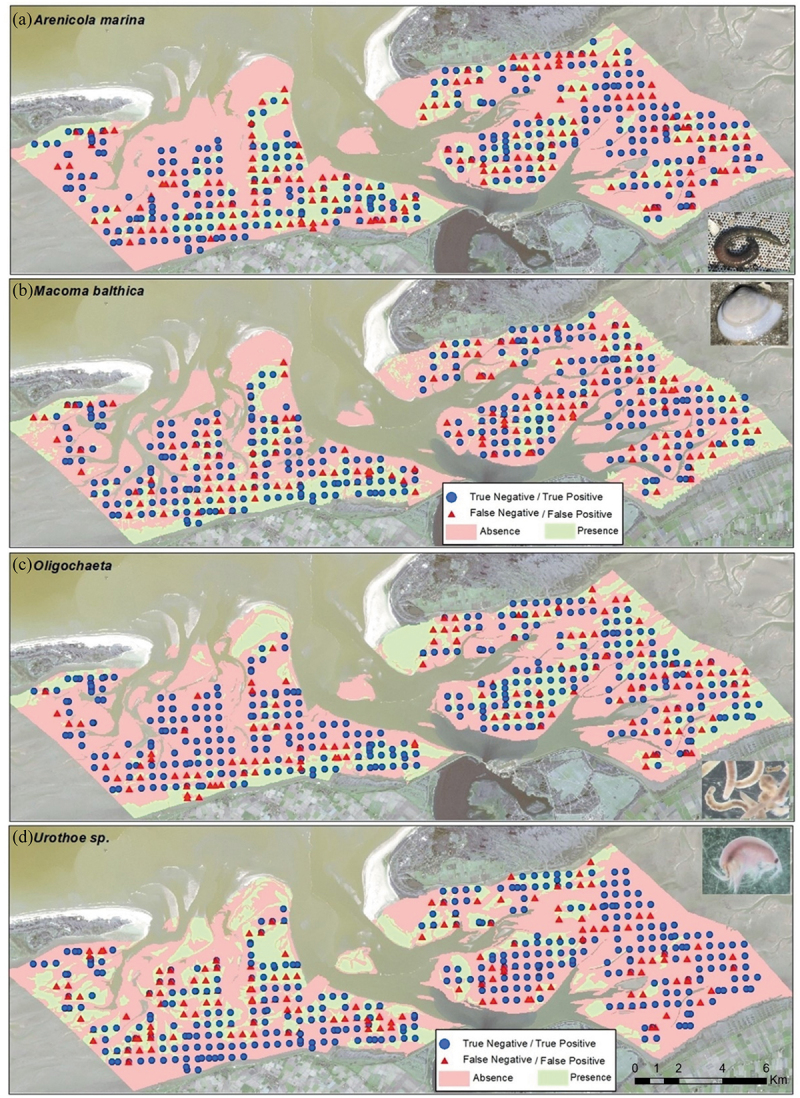
Predicted distribution maps of the four species with best object size for each species (Table 5) for 2019: (a) *Arenicola marina* (13 ha), (b) *Macoma balthica* (0.3 ha), (c) *Oligochaeta* (3.5 ha), and (d) *Urothoe sp*. (11 ha). Pictures of the species were taken during field and lab work by Logambal Madhuanand.

Predictive maps of the study area were generated for the four variables (Figure 7) and the species distribution (Figure 8) using the chosen best object set with the corresponding object size.

The patterns observed in the predictions were in line with the expectations for both the tidal basins. The inverse relation between median grain size and silt content is evident in the predictive maps. The sediment characteristics differed between the two basins with an overall shifting pattern moving from the mainland coast (south) towards the sea (north). Also, the predictions of biomass and species richness in low energetic zones, particularly near the coast and away from the tidal inlets where they are typically found, were clearly visible on the maps.

Descriptive statistics of the predicted objects from the spatial maps were compared to those of the field-sampled points for the four variables during the same year (Table 6). Median grain size, silt content, and species richness were predicted well and matched closer to the field observations than those of the biomass predictions. The standard deviations for the predicted objects were notably lower than those for the field observations that exhibited larger variability, while their medians remained closer. By analysing the range between the minimum and maximum values of the predicted objects, it was also clear that the model was capable of capturing extreme values for most of these variables except for biomass.

**Table 6.** Descriptive statistics of the four environmental variables for the field points and all predicted objects for 2019.

Variables	Field points				Predicted Objects			
	Median	Std	Min-Max	No. of points	Median	Std	Min-Max	No. of points
Median grain size (µm)	148	36.8	21-222	462	152	24.3	61-202	1320
Silt content (%)	10.1	14.4	1.25-78.1	462	7.18	7.07	2.33-47.3	7512
Biomass (gAFDM/m^2^)	10.6	49.5	0.01-684.1	462	2.8	5.3	0.18-82.3	5820
Species richness (no. of species/sample)	8	3.15	1-18	462	6.2	1.5	3-13	7512

Presence/absence distribution maps were created for the four species units using the chosen object set and object area based on prediction accuracy (Figure 8).

All predicted species distributions pointed to concentrations near the coast and away from the tidal inlets as observed with the field points. *Macoma balthica* and *Oligochaeta* were present in fine-grained material compared to the other two species, as evidenced by their presence in specific locations and the corresponding sediment characteristics within those regions (Figure 7). The misclassifications (Figure 8) were uniformly distributed across various regions for all four predicted species.

## 4. Discussion

A combination of OBIA and deep learning features was used to predict the distribution of the macrozoobenthos properties and sediment properties on tidal flats. The segmentation process involved merging the pixels of the tidal flats into objects that matched the expected landscape patterns, like delineating the channels. These objects adhered to the spatial structure of the tidal flats. Although all four variables were collected at the same sampling stations, the optimal object scales and input properties differed. Identification of proper segmentation parameters was crucial for OBIA and often was hindered by the spectral homogeneity of the tidal flats.

### 4.1. Benefits of OBIA

The object-based model performed better than the pixel-based approach for both environmental variables and species distributions. The benefits of using Object-Based Image Analysis (OBIA) were evident, with a 21% point increase in accuracy over the pixel-based model using only spectral bands. When comparing the results to those of Madhuanand et al. (2023), who showed that their best results were achieved using a pixel-based approach with spectral bands and deep-learning features, our new model, which employed the object-based approach incorporating both spectral bands and deep learning features, exhibited an average improvement of 7% points in R^2^. Unlike the pixel-based method that centred on average values, our new model also better captured extreme values (Table 6). On observing the object set d), which incorporated spectral bands and deep-learning features, with object set b), which included spectral bands and textural features, it is evident that both of these sets included spatial, spectral, and textural information. It was the deep-learning features that consistently exhibited better performance across all variables, surpassing the textural properties derived from OBIA. These deep-learning features contained information obtained through filters that were far more effective than the textural and geometrical properties derived from OBIA. Incorporating information from neighbouring pixels into the analysis through objects not only enhanced the richness of features but also mitigated the uncertainties associated with using pixel information in isolation. Thus OBIA facilitated the integration of several data sources that improved the prediction accuracies and proved to overcome the pixel-based approach for this application. The significance of the spatial aspect in relation to these environmental variables was also evident.

### 4.2. Sensitivity to object size

The prediction scores showed a clear dependency on object size implying the sensitivity of the predictions to the spatial support scale of remote sensing data. The optimal spatial units of all the four variables, median grain size, silt content, biomass, and species richness were between 2.0 and 12 ha, smaller than those of the object sizes defined with spectral bands (Figure A2). The shape and compactness pertained to smooth border shapes and promote object compactness, exhibited minimal influence on the prediction scores concerning the scale. The median grain size predictions were best compared to the other three variables. The object size with the best prediction results was considerably smaller than the sample variogram range (Figure 3) for each of the predicted environmental variables, and a longer variogram range was associated with a larger optimal object. This association is also reflected in the drop in model performance for objects larger than the range size as visible in Figure 5, where the length of an object can be approximated by the square root of its area. This difference in optimal object sizes reinforces the importance of considering the spatial scale when analysing the environmental variables.

Similarly, the species distribution models revealed significant variations in object size across the four selected species ranging from 0.3 ha to 13 ha. *Macoma balthica* and *Oligochaeta* exhibited high prediction scores in cases where smaller object sizes were employed, indicating localized variations among adjacent data points, with the spatial component playing a substantial role. Specifically, in the case of *Macoma balthica*, the model’s smaller object size aligned with the smaller range of the variogram (Figure A1). Conversely, for *Oligochaeta*, a slightly larger range was favoured, corresponding to an object size larger than that for *Macoma*. While examining the presence/absence patterns of *Arenicola marina* and *Urothoe sp.*, spatial autocorrelation was detected, and these patterns exhibited less variability in adjacent points resulting in larger object sizes. For the prediction of individual species (groups), a clear relation between object size and variogram range was not apparent.

### 4.3. Object properties

The object sets defined with spectral bands and deep-learning features showed consistently better prediction scores for all variables than using just the spectral bands. The four variables were predicted with an R^2^ score ranging from 0.31 (for macrozoobenthic biomass) to 0.54 (for median grain size) (Table A3). Prediction accuracy values for all four variables showed that including spectral bands and deep learning features yielded optimal results. The inclusion of additional properties such as texture and geometry did not enhance the results.

The distribution of the four species was predicted well with F1 scores ranging from 0.54 (*Arenicola marina*) to 0.68 (*Macoma balthica*) (Table A4). These species exist both on the surface and up to 30 cm below the subsurface which adds to the challenge of mapping. The environmental conditions, the resolution of the remote-sensing imagery, and the complexity of the landscape all play a role in mapping the distribution of species. For each species, predictions were better when using the spectral bands and features, although the optimal object size varied.

From the predictive maps, we observed that all variables were predicted well in line with the expectations. The inverse relation between median grain size and silt content, as well as the differences in sediment pattern between the two basins were captured by the prediction models. The biomass and species richness predictions showed higher uncertainty with a tendency to fit towards the mean (Table A3; Table 6), in other words, extreme value predictions were difficult. The species distribution maps were predicted well especially the species presence was aligned in areas with high biomass and species richness predictions. This indicates the model consistency over the environmental variables and species distributions.

Compton et al. (2013) determined the functional linkages with the benthic organisms with six environmental variables such as sediment, exposure time, microphytobenthic organisms, salinity, tidal currents and orbital velocities. Furthermore, Wal and Herman (2007) demonstrated that a combination of microwave and optical remote sensing images is essential to reliably map sediment characteristics. While our model yielded accuracy levels comparable to those presented by Wal and Herman (2007) concerning environmental variables, median grain size, and silt content in a similar inter-tidal setting, leveraging on spectral bands and features derived from the deep learning model. Notably, these features are more accessible than microwave images, highlighting the practicality and efficiency of our approach. However, the complexity of our deep learning model approach limits the accessibility compared to the models used in those studies.

In a recent study on microphytobenthic biomass, Zhang et al. (2023) achieved an R^2^ of 0.59 with a combination of in-situ and Sentinel-2 data over an estuarine tidal flat region using a pixel-based approach. This result closely aligns with our model’s estimation of 0.54, which was obtained in a dynamic environment that included spatial information. Total biomass and species richness were correlated with NDVI, in conjunction with in-situ measurements, by Van der Wal et al. (2008) resulting in R^2^ values of 0.40 and 0.43, respectively. While maintaining broad applicability across diverse regions through the utilization of openly accessible remote sensing data, our OBIA model, with the use of spectral bands and extracted features from a single image, achieved a prediction score of 0.31 and 0.35 for biomass and species richness, respectively.

### 4.4. Period between field observations and image acquisition

Ideally, field observations and image acquisition would all occur on a single day. While the field observations cover up to a month, image availability is steered by cloud cover and tidal exposure. Image availability at useably low tide levels is, however, limited, leading to the current selection (Table 1). As a consequence, the time gap between the field and image dates is several months. The tidal-induced variation in sediment transport, sedimentation, and resuspension is dynamic and especially the diurnal dynamics in the intertidal areas are large. However, Folmer et al. (2023) analysed field data on sediment composition in intertidal systems and found synchronized patterns in sediment composition between 2009 and 2019. They observed an overall consistent distribution in sediment composition within the studied period. Hence, we expect the impacts of the time gap between field and image data on the model training and prediction results to be relatively small.

While deep learning methods could be advantageous for direct prediction, they typically require more training data than random forest regressors to achieve optimal performance. We integrated a deep learning feature extraction model with an Object-Based Image Analysis (OBIA) approach that leverages a larger volume of imagery through unsupervised feature extraction. Combined with the spatial strengths of OBIA, the features from Sentinel-2 imagery amplify the predictive power. As a result, the predictions of environmental variables and species distributions across the two regions over 3 years were improved using OBIA and deep-learning features.

## 5. Conclusions

In this study, the prediction accuracies of the three environmental indicators: sediment, macrozoobenthos biomass, and species distribution on tidal flats were evaluated when deep-learning features were combined with an object-based image analysis model. We created sets of objects using spectral bands and/or deep-learning features and then incorporated image-derived properties that describe these objects. The resulting five object sets, along with field points, were utilized to train a random forest model, aimed at predicting environmental variables. The model was able to predict the three indicators for different object sizes and properties. The object set with spectral bands and deep-learning features consistently outperformed the other object sets for the sediment characteristics, macrozoobenthic properties, and species distributions. The optimal object size differed for the four variables: median grain size, silt content, biomass, and species richness and for the species distributions, between 0.3 ha and 13 ha. The R^2^ score for the four variables ranged between 0.31 (for macrozoobenthic biomass) and 0.54 (for median grain size), while the F1 scores for the distribution of the four species ranged from 0.54 (*Arenicola marina*) to 0.68 (*Macoma balthica*). The combination of spectral bands and deep-learning features consistently showed strong performance across all variables. For the four environmental variables, predictions made for objects based on spectral bands and deep-learning features showed an average improvement of 8.9% points compared to predictions for objects based on spectral bands only. Also, there was an average improvement of 7% points when comparing pixel-based approach with spectral bands and deep-learning features to that of the OBIA model with the same input object set. Overall, the object-based method with features improved prediction accuracy by 21% points compared to per-pixel-based image analysis using just spectral bands on average for the four variables. Despite the increased number of parameters, this approach with deep-learning features and OBIA is an effective way to map variables in regions with lower spectral contrast, such as tidal flats, using remote sensing images. By applying OBIA along with deep learning features, researchers and coastal managers can enhance their understanding of tidal flat dynamics, support conservation efforts, and make informed decisions regarding coastal management and planning.

## Supplementary Material

OBIA_lfs_Supplementary_09_09_2024.docx

## Data Availability

All source code of this research and data will be made available at https://github.com/logsanand/Featureextraction-OBIA.

## References

[cit0001] Addink, E. A., S. M. De Jong, and E. J. Pebesma. 2007. “The Importance of Scale in Object-Based Mapping of Vegetation Parameters with Hyperspectral Imagery.” *Photogrammetric Engineering & Remote Sensing* 73 (8): 905–912. 10.14358/PERS.73.8.905.

[cit0002] Addink, E. A., F. M. B. Van Coillie, and S. M. de Jong. 2012. “Introduction to the GEOBIA 2010 Special Issue: From Pixels to Geographic Objects in Remote Sensing Image Analysis.” *International Journal of Applied Earth Observation and Geoinformation* 15 (1): 1–6. 10.1016/j.jag.2011.12.001.

[cit0003] Andersen, T. J., and M. Pejrup. 2001. “Suspended Sediment Transport on a Temperate, Microtidal Mudflat, the Danish Wadden Sea.” *Marine Geology* 173 (1–4): 69–85. 10.1016/S0025-3227(00)00164-X.

[cit0004] Baatz, M., and S. Arno. 2000. “Multiresolution Segmentation: An Optimization Approach for High Quality Multi-Scale Image Segmentation.” In *Proceedings of Angewandte Geographische Informationsverarbeitung XII* Salzburg, 12–23.

[cit0005] Barale, V., and M. Gade. 2018. *Remote Sensing of the Asian Seas*, Springer International Publishing. 10.1007/978-3-319-94067-0.

[cit0006] Benz, U. C., P. Hofmann, G. Willhauck, I. Lingenfelder, and M. Heynen. 2004. “Multi-Resolution, Object-Oriented Fuzzy Analysis of Remote Sensing Data for GIS-Ready Information.” *Isprs Journal of Photogrammetry & Remote Sensing* 58 (3–4): 239–258. 10.1016/j.isprsjprs.2003.10.002.

[cit0007] Beukema, J. J. 1976. “Biomass and Species Richness of the Macro-Benthic Animals Living on the Tidal Flats of the Dutch Wadden Sea.” *Netherlands Journal of Sea Research* 10 (2): 236–261. 10.1016/0077-7579(76)90017-X.

[cit0008] Bijleveld, A. I., J. A. van Gils, J. van der Meer, A. Dekinga, C. Kraan, H. W. van der Veer, and T. Piersma. 2012. “Designing a Benthic Monitoring Programme with Multiple Conflicting Objectives.” *Methods in Ecology and Evolution* 3 (3): 526–536. 10.1111/j.2041-210X.2012.00192.x.

[cit0009] Blaschke, T. 2010. “Object Based Image Analysis for Remote Sensing.” *Isprs Journal of Photogrammetry & Remote Sensing* 65 (1): 2–16. 10.1016/j.isprsjprs.2009.06.004. PMC3945831 24623958

[cit0010] Blaschke, T., G. J. Hay, M. Kelly, S. Lang, P. Hofmann, E. Addink, R. Queiroz Feitosa, et al. 2014. “Geographic Object-Based Image Analysis - Towards a New Paradigm.” *Isprs Journal of Photogrammetry & Remote Sensing* 87:180–191. 10.1016/j.isprsjprs.2013.09.014. 24623958 PMC3945831

[cit0011] Breiman, L. 2001. “Random Forests.” *Machine Learning* 45 (1): 5–32. 10.1007/978-3-030-62008-0_35.

[cit0012] Burnett, C., and T. Blaschke. 2003. “A Multi-Scale Segmentation/Object Relationship Modelling Methodology for Landscape Analysis.” *Ecological Modelling* 168 (3): 233–249. 10.1016/S0304-3800(03)00139-X.

[cit0013] Chinchor, N. 1992. “MUC-4 Evaluation Metrics.” In *4th Message Understanding Conference, MUC 1992 - Proceedings* Virginia, 22–29. 10.3115/1072064.1072067.

[cit0014] Compton, T. J., S. Holthuijsen, A. Koolhaas, A. Dekinga, J. ten Horn, J. Smith, Y. Galama, et al. 2013. “Distinctly Variable Mudscapes: Distribution Gradients of Intertidal Macrofauna Across the Dutch Wadden Sea.” *Journal of Sea Research* 82:103–116. 10.1016/j.seares.2013.02.002.

[cit0015] Curran, P. J. 1988. “The Semivariogram in Remote Sensing: An Introduction.” *Remote Sensing of Environment* 24 (3): 493–507. 10.1016/0034-4257(88)90021-1.

[cit0016] Drent, J., R. Bijkerk, M. Herlyn, M. Grotjahn, J. Voß, M.-C. Carausu, and D. W. Thieltges. 2017. Wadden Sea Quality Status Report - Alien Species. *Wadden Sea Quality Status Report 2017*. https://qsr.waddensea-worldheritage.org/reports/alien-species.

[cit0017] Folmer, E., A. Dekinga, S. Holthuijsen, J. van der Meer, D. Mosk, T. Piersma, and H. van der Veer. 2017 Species Distribution Models of Intertidal Benthos : tools for Assessing the Impact of Physical and Morphological Drivers on Benthos and Birds in the Wadden Sea Introduction The Wadden Sea and its coastal area is threaten 753288 (Netherlands: NIOZ) https://open.rijkswaterstaat.nl/open-overheid/@48392/species-distribution-models-intertidal/.

[cit0018] **114 p. Ill., app. With ref**.

[cit0019] Folmer, E. O., A. I. Bijleveld, S. Holthuijsen, J. van der Meer, T. Piersma, and H. W. van der Veer. 2023. “Space–Time Analyses of Sediment Composition Reveals Synchronized Dynamics at All Intertidal Flats in the Dutch Wadden Sea.” *Estuarine, Coastal and Shelf Science* 285 (February): 108308. 10.1016/j.ecss.2023.108308.

[cit0020] Haralick, R. M., K. Sam Shanmugam, and I. Dinstein. 1973. “Textural Features for Image Classification.” *IEEE Transactions on Systems, Man, and Cybernetics* 3 (6): 610–621. 10.1190/segam2015-5927230.1.

[cit0021] Hay, G. J., and G. Castilla. 2008. “Geographic Object-Based Image Analysis (GEOBIA): A New Name for a New Discipline.” In *Object-Based Image Analysis: Spatial Concepts for Knowledge-Driven Remote Sensing Applications, Lecture Notes in Geoinformation and Cartography*, edited by T. Blaschke, S. Lang, and G. Hay, 75–89. Berlin Heidelberg: Springer. 10.1007/978-3-540-77058-9_4.

[cit0022] Herman, P. M. J., J. J. Middelburg, J. van de Koppel, and C. H. R. Heip. 1999. “Ecology of Estuarine Macrobenthos.” In *Advances in Ecological Research*, Vol. 29. Elsevier Masson SAS. 10.1016/S0065-2504(08)60194-4.

[cit0023] Jung, Y., and J. Hu. 2015. “A K -Fold Averaging Cross-Validation Procedure.” *Journal of Nonparametric Statistics* 27 (2): 167–179. 10.1080/10485252.2015.1010532. 27630515 PMC5019184

[cit0024] Kirwan, M. L., and J. Patrick Megonigal. 2013. “Tidal Wetland Stability in the Face of Human Impacts and Sea-Level Rise.” *Nature* 504 (7478): 53–60. 10.1038/nature12856. 24305148

[cit0025] Kuipers, B. R., P. A. W. J. de Wilde, and F. Creutzberg. 1981. “Energy Flow in a Tidal Flat Ecosystem.” *Marine Ecology Progress Series* 5:215–221. 10.3354/meps005215.

[cit0026] Lyashevska, O., D. J. Brus, and J. van der Meer. 2016. “Grid-Spacing and the Quality of Abundance Maps for Species That Show Spatial Autocorrelation and Zero-Inflation.” *Spatial Statistics* 18:386–395. 10.1016/j.spasta.2016.08.001.

[cit0027] Ma, L., M. Li, X. Ma, L. Cheng, P. Du, and Y. Liu. 2017. “A Review of Supervised Object-Based Land-Cover Image Classification.” *Isprs Journal of Photogrammetry & Remote Sensing* 130:277–293. 10.1016/j.isprsjprs.2017.06.001.

[cit0028] Madhuanand, L., C. J. M. Philippart, J. Wang, W. Nijland, S. M. de Jong, A. I. Bijleveld, and E. A. Addink. 2023. “Enhancing the Predictive Performance of Remote Sensing for Ecological Variables of Tidal Flats Using Encoded Features from a Deep Learning Model.” *GIScience & Remote Sensing* 60 (1). 10.1080/15481603.2022.2163048.

[cit0029] Mao, D., Z. Wang, B. Du, L. Li, Y. Tian, M. Jia, Y. Zeng, K. Song, M. Jiang, and Y. Wang. 2020. “National Wetland Mapping in China: A New Product Resulting from Object-Based and Hierarchical Classification of Landsat 8 OLI Images.” *Isprs Journal of Photogrammetry & Remote Sensing* 164 (March): 11–25. 10.1016/j.isprsjprs.2020.03.020.

[cit0030] Monteiro, J. G., J. L. Jiménez, F. Gizzi, P. Přikryl, J. S. Lefcheck, R. S. Santos, and J. Canning-Clode. 2021. “Novel Approach to Enhance Coastal Habitat and Biotope Mapping with Drone Aerial Imagery Analysis.” In *Scientific Reports*, Vol. 11. Nature Publishing Group UK. 10.1038/s41598-020-80612-7. PMC7804263 33436894

[cit0031] Murray, N. J., S. R. Phinn, M. DeWitt, R. Ferrari, R. Johnston, M. B. Lyons, N. Clinton, D. Thau, and R. A. Fuller. 2019. “The Global Distribution and Trajectory of Tidal Flats.” *Nature* 565 (7738): 222–225. 10.1038/s41586-018-0805-8. 30568300

[cit0032] Nijland, W., E. A. Addink, S. M. De Jong, and D. Van der Meer. 2009. “Optimizing Spatial Image Support for Quantitative Mapping of Natural Vegetation.” *Remote Sensing of Environment* 113 (4): 771–780. 10.1016/j.rse.2008.12.002.

[cit0033] Phinn, S. R., C. M. Roelfsema, and P. J. Mumby. 2012. “Multi-Scale, Object-Based Image Analysis for Mapping Geomorphic and Ecological Zones on Coral Reefs.” *International Journal of Remote Sensing* 33 (12): 3768–3797. 10.1080/01431161.2011.633122.

[cit0034] Reise, K. 1985. “Tidal Flat Ecology.” In *Ecological Studies*, 1–2. Springer Study Edition. 10.1007/978-3-642-70495-6_1.

[cit0035] Rijkswaterstaat. n.d. “Getij | Rijkswaterstaat.” Accessed November 1, 2021. https://www.rijkswaterstaat.nl/water/waterdata-en-waterberichtgeving/waterdata/getij.

[cit0036] Roelfsema, C., E. Kovacs, J. Carlos Ortiz, N. H. Wolff, D. Callaghan, M. Wettle, M. Ronan, S. M. Hamylton, P. J. Mumby, and S. Phinn. 2018. “Coral Reef Habitat Mapping: A Combination of Object-Based Image Analysis and Ecological Modelling.” *Remote Sensing of Environment* 208:27–41. 10.1016/j.rse.2018.02.005.

[cit0037] Simonyan, K., A. Vedaldi, and A. Zisserman. 2014. “Deep Inside Convolutional Networks: Visualising Image Classification Models and Saliency Maps.” *2nd International Conference on Learning Representations, ICLR 2014 - Workshop Track Proceedings* Banff, AB, Canada, 1–8.

[cit0038] Tian, B., Y. Zhou, L. Zhang, and L. Yuan. 2008. “Analyzing the Habitat Suitability for Migratory Birds at the Chongming Dongtan Nature Reserve in Shanghai, China.” *Estuarine, Coastal and Shelf Science* 80 (2): 296–302. 10.1016/j.ecss.2008.08.014.

[cit0039] Trimble. n.d. “ECognition (V9).” Manufactured by Trimble Inc. 935 Stewart Drive Sunnyvale, CA 94085, USA, https://geospatial.trimble.com/products-and-solutions/trimble-ecognition.

[cit0040] User Guides. 2015. “User Guides - Sentinel-2 MSI.” ESA-Sentinel 2. 2015. https://sentinel.esa.int/web/sentinel/user-guides/sentinel-2-msi.

[cit0041] Wal, D. van der, and P.M. J. Herman. 2007. “Regression-Based Synergy of Optical, Shortwave Infrared and Microwave Remote Sensing for Monitoring the Grain-Size of Intertidal Sediments.” *Remote Sensing of Environment* 111 (1): 89–106. 10.1016/j.rse.2007.03.019.

[cit0042] Wal, D. van der, P. M. J. Herman, R. M. Forster, T. Ysebaert, F. Rossi, E. Knaeps, Y. M. G. Plancke, and S. J. Ides. 2008. “Distribution and Dynamics of Intertidal Macrobenthos Predicted from Remote Sensing: Response to Microphytobenthos and Environment.” *Marine Ecology Progress Series* 367:57–72. 10.3354/meps07535.

[cit0043] Wolff, W. J. 2013. “Ecology of the Wadden Sea: Research in the Past and Challenges for the Future.” *Journal of Sea Research* 82:3–9. 10.1016/j.seares.2013.03.006.

[cit0044] Zhang, T., B. Tian, Y. Wang, D. Liu, Y. Zhou, and D. van der Wal. 2023. “Mapping Depth-Integrated Microphytobenthic Biomass on an Estuarine Tidal Flat Using Sentinel Satellite Data.” *International Journal of Applied Earth Observation and Geoinformation* 122:103417. 10.1016/j.jag.2023.103417.

