# Supplementary material for: Optimizing predictions of environmental variables and species distributions on tidal flats by combining Sentinel-2 images and their deep-learning features with OBIA
Source: Int J Remote Sens. 2024 Nov 19;46(2):811–34. doi: 10.1080/01431161.2024.2423909 (PMC11755323; doi:10.1080/01431161.2024.2423909)
Supplement: OBIA_lfs_Supplementary_09_09_2024.docx [file TRES_A_2423909_SM5551.docx]

**APPENDIX**

Figure A1. Semivariograms of the four environmental variables for three years and two regions combined. (*a*) *Arenicola marina* (*b*) *Macoma balthica* (*c*) *Oligochaeta* (*d*) *Urothoe sp.*


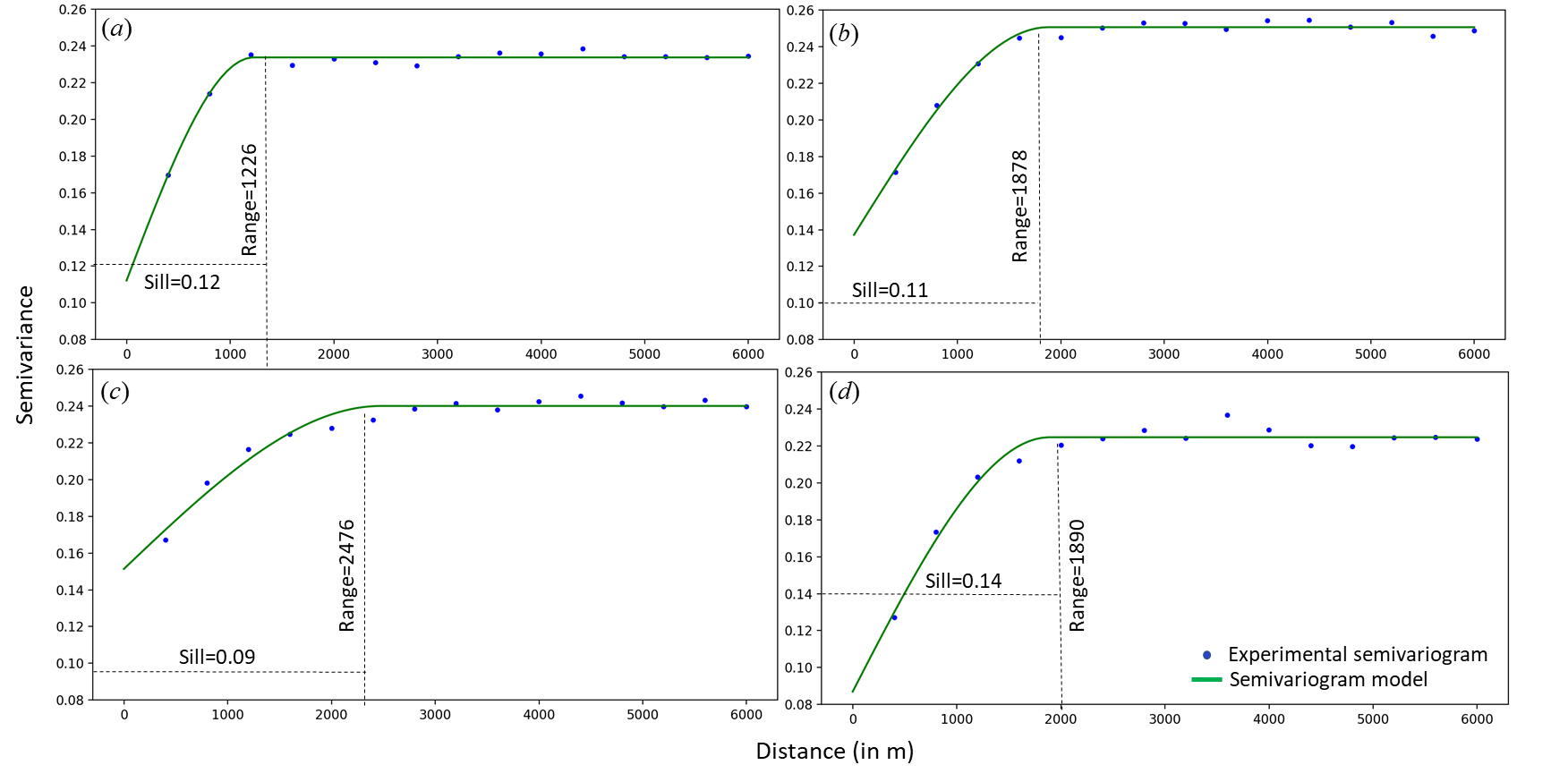


## Object sizes

Object sets were successfully defined from the spectral and deep-learning features combination for the scale values 10-200 and the chosen object properties. The average object sizes generated for sets a) and b) (using only spectral bands) ranged from 0.31 ha to 34 ha, while for sets c), d) and e) (using spectral bands along with features or only features) the sizes were smaller with average object sizes ranged from 0.07 ha to 15 ha (Figure A2). As the scale increased, the total number of objects decreased from 199109 to 1787 for the objects with just the spectral bands and from 838410 to 4169 for the object set with both spectral bands and features or only features.


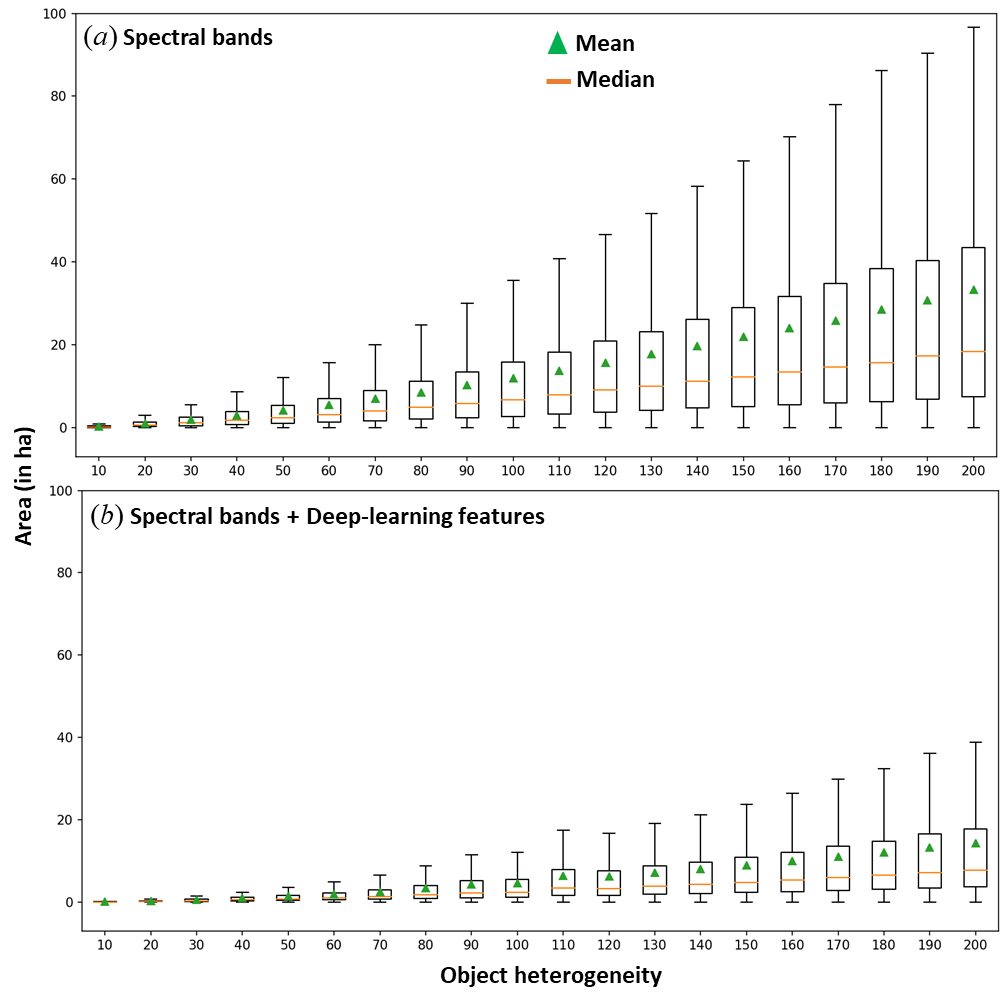


Figure A2. Object sizes with different scale settings A) Spectral bands and B) Spectral + deep learning feature bands. The box in the plot shows 25^th^ and 75^th^ quartile, whiskers drawn within 1.5 times the interquartile range, along with median & mean.

Table A3. Prediction scores of all four variables for five object sets for the scale values 10-200

|  | Median grain size  (µm) | | | | | Silt content  (% of volume <63µm) | | | | | Biomass  (gADW/m^2^) | | | | | Species richness  (no. of species/sample) | | | | |
| --- | --- | --- | --- | --- | --- | --- | --- | --- | --- | --- | --- | --- | --- | --- | --- | --- | --- | --- | --- | --- |
| Scale | **A** | **B** | **C** | **D** | **E** | **A** | **B** | **C** | **D** | **E** | **A** | **B** | **C** | **D** | **E** | **A** | **B** | **C** | **D** | **E** |
| 10 | 0.356 | 0.308 | 0.403 | 0.411 | 0.391 | 0.387 | 0.340 | 0.468 | 0.469 | 0.444 | 0.203 | 0.202 | 0.263 | 0.271 | 0.258 | 0.221 | 0.205 | 0.275 | 0.284 | 0.268 |
| 20 | 0.379 | 0.337 | 0.432 | 0.445 | 0.423 | 0.407 | 0.379 | 0.480 | 0.494 | 0.465 | 0.229 | 0.229 | 0.267 | 0.272 | 0.258 | 0.218 | 0.215 | 0.295 | 0.296 | 0.285 |
| 30 | 0.375 | 0.348 | 0.449 | 0.448 | 0.436 | 0.411 | 0.375 | 0.493 | 0.488 | 0.475 | 0.224 | 0.255 | 0.281 | 0.291 | 0.285 | 0.224 | 0.228 | 0.289 | 0.318 | 0.309 |
| 40 | 0.398 | 0.361 | 0.469 | 0.463 | 0.448 | 0.415 | 0.391 | 0.496 | 0.485 | 0.469 | 0.215 | 0.257 | 0.291 | 0.284 | 0.273 | 0.236 | 0.232 | 0.293 | 0.313 | 0.308 |
| 50 | 0.431 | 0.391 | 0.484 | 0.479 | 0.465 | 0.414 | 0.387 | 0.501 | 0.493 | 0.483 | 0.263 | 0.266 | 0.296 | 0.275 | 0.286 | 0.251 | 0.252 | 0.301 | 0.333 | 0.333 |
| 60 | 0.434 | 0.411 | 0.508 | 0.488 | 0.477 | 0.423 | 0.401 | **0.509** | 0.496 | 0.476 | 0.241 | 0.238 | 0.279 | 0.305 | 0.303 | 0.259 | 0.259 | 0.317 | **0.357** | 0.347 |
| 70 | 0.449 | 0.437 | 0.523 | 0.488 | 0.481 | 0.448 | 0.422 | 0.508 | 0.488 | 0.482 | 0.233 | 0.249 | 0.263 | **0.311** | 0.298 | 0.270 | 0.279 | 0.301 | 0.354 | 0.345 |
| 80 | 0.476 | 0.448 | 0.498 | 0.497 | 0.474 | 0.450 | 0.430 | 0.487 | 0.484 | 0.463 | 0.248 | 0.248 | 0.283 | 0.293 | 0.279 | 0.281 | 0.274 | 0.322 | 0.343 | 0.342 |
| 90 | 0.496 | 0.478 | 0.503 | 0.495 | 0.488 | 0.451 | 0.442 | 0.482 | 0.479 | 0.468 | 0.219 | 0.219 | 0.264 | 0.280 | 0.279 | 0.279 | 0.275 | 0.313 | 0.302 | 0.329 |
| 100 | 0.497 | 0.492 | 0.493 | 0.502 | 0.509 | 0.428 | 0.417 | 0.477 | 0.478 | 0.481 | 0.216 | 0.218 | 0.265 | 0.261 | 0.263 | 0.278 | 0.274 | 0.317 | 0.304 | 0.310 |
| 110 | 0.496 | 0.492 | 0.496 | 0.491 | 0.481 | 0.427 | 0.421 | 0.469 | 0.474 | 0.459 | 0.221 | 0.217 | 0.266 | 0.254 | 0.253 | 0.269 | 0.270 | 0.327 | 0.299 | 0.302 |
| 120 | 0.489 | 0.496 | 0.486 | 0.499 | 0.492 | 0.405 | 0.403 | 0.469 | 0.456 | 0.459 | 0.184 | 0.187 | 0.259 | 0.261 | 0.259 | 0.266 | 0.249 | 0.324 | 0.297 | 0.297 |
| 130 | 0.494 | 0.494 | 0.504 | 0.511 | 0.507 | 0.401 | 0.383 | 0.475 | 0.459 | 0.460 | 0.170 | 0.175 | 0.255 | 0.255 | 0.253 | 0.261 | 0.255 | 0.305 | 0.298 | 0.296 |
| 140 | 0.497 | 0.492 | 0.510 | 0.518 | 0.516 | 0.393 | 0.382 | 0.482 | 0.471 | 0.471 | 0.187 | 0.169 | 0.225 | 0.252 | 0.245 | 0.281 | 0.260 | 0.294 | 0.297 | 0.302 |
| 150 | 0.494 | 0.482 | 0.517 | 0.507 | 0.503 | 0.399 | 0.388 | 0.480 | 0.464 | 0.457 | 0.149 | 0.137 | 0.222 | 0.249 | 0.255 | 0.268 | 0.248 | 0.278 | 0.290 | 0.289 |
| 160 | 0.505 | 0.493 | 0.528 | 0.516 | 0.509 | 0.392 | 0.385 | 0.489 | 0.451 | 0.441 | 0.135 | 0.117 | 0.217 | 0.223 | 0.235 | 0.249 | 0.236 | 0.282 | 0.273 | 0.271 |
| 170 | 0.508 | 0.505 | 0.521 | 0.525 | 0.517 | 0.392 | 0.383 | 0.492 | 0.446 | 0.437 | 0.142 | 0.131 | 0.209 | 0.206 | 0.219 | 0.246 | 0.233 | 0.275 | 0.257 | 0.256 |
| 180 | 0.509 | 0.502 | 0.534 | **0.535** | 0.522 | 0.394 | 0.386 | 0.489 | 0.442 | 0.432 | 0.141 | 0.126 | 0.210 | 0.208 | 0.209 | 0.246 | 0.228 | 0.272 | 0.256 | 0.256 |
| 190 | 0.515 | 0.506 | 0.525 | 0.519 | 0.509 | 0.390 | 0.381 | 0.461 | 0.418 | 0.407 | 0.149 | 0.128 | 0.194 | 0.208 | 0.208 | 0.244 | 0.235 | 0.266 | 0.250 | 0.246 |
| 200 | 0.507 | 0.496 | 0.531 | 0.516 | 0.511 | 0.385 | 0.376 | 0.444 | 0.410 | 0.402 | 0.159 | 0.137 | 0.195 | 0.203 | 0.205 | 0.245 | 0.235 | 0.253 | 0.250 | 0.248 |
|  | **A) Spectral bands (SP)**  **B) Spectral bands + Texture +Geo (SP + TXT + GEO)** | | | | | | **C) Deep learning features (FEA)**  **D) Deep learning features + Spectral bands (FEA +SP)** | | | | | | | **E) Deep learning features + Spectral bands + Texture + Geometry (FEA + SP +TXT +GEO)** | | | | | | |

Table A4. F1 scores of all four species for five object sets for the scale values 10-200

|  | Arenicola marina | | | | | Macoma balthica | | | | | Oligochaeta | | | | | Urothoe sp. | | | | |
| --- | --- | --- | --- | --- | --- | --- | --- | --- | --- | --- | --- | --- | --- | --- | --- | --- | --- | --- | --- | --- |
| Scale | **A** | **B** | **C** | **D** | **E** | **A** | **B** | **C** | **D** | **E** | **A** | **B** | **C** | **D** | **E** | **A** | **B** | **C** | **D** | **E** |
| 10 | 0.501 | 0.425 | 0.480 | 0.501 | 0.463 | 0.653 | 0.627 | 0.665 | 0.674 | 0.659 | 0.542 | 0.540 | 0.573 | 0.578 | 0.584 | 0.457 | 0.432 | 0.550 | 0.502 | 0.487 |
| 20 | 0.490 | 0.467 | 0.478 | 0.504 | 0.501 | 0.663 | 0.627 | 0.673 | **0.682** | 0.664 | 0.549 | 0.558 | 0.590 | 0.591 | 0.581 | 0.477 | 0.454 | 0.517 | 0.551 | 0.524 |
| 30 | 0.493 | 0.449 | 0.504 | 0.494 | 0.484 | 0.654 | 0.650 | 0.669 | 0.677 | 0.659 | 0.537 | 0.573 | 0.599 | 0.609 | 0.612 | 0.479 | 0.461 | 0.540 | 0.548 | 0.529 |
| 40 | 0.489 | 0.457 | 0.518 | 0.499 | 0.483 | 0.639 | 0.642 | 0.681 | 0.675 | 0.649 | 0.541 | 0.574 | 0.580 | 0.612 | 0.601 | 0.457 | 0.467 | 0.533 | 0.552 | 0.519 |
| 50 | 0.489 | 0.466 | 0.522 | 0.517 | 0.504 | 0.664 | 0.642 | 0.669 | 0.676 | 0.646 | 0.583 | 0.582 | 0.599 | 0.614 | 0.616 | 0.485 | 0.479 | 0.543 | 0.538 | 0.518 |
| 60 | 0.477 | 0.478 | 0.505 | 0.521 | 0.507 | 0.663 | 0.660 | 0.663 | 0.675 | 0.648 | 0.583 | 0.580 | 0.604 | 0.616 | 0.636 | 0.486 | 0.497 | 0.531 | 0.526 | 0.504 |
| 70 | 0.490 | 0.495 | 0.507 | 0.498 | 0.493 | 0.674 | 0.656 | 0.674 | 0.669 | 0.648 | 0.584 | 0.603 | 0.599 | 0.624 | 0.630 | 0.467 | 0.510 | 0.522 | 0.519 | 0.513 |
| 80 | 0.528 | 0.499 | 0.500 | 0.509 | 0.473 | 0.665 | 0.645 | 0.658 | 0.669 | 0.647 | 0.559 | 0.577 | 0.617 | 0.633 | **0.642** | 0.485 | 0.501 | 0.533 | 0.523 | 0.519 |
| 90 | 0.516 | 0.497 | 0.503 | 0.525 | 0.485 | 0.643 | 0.623 | 0.659 | 0.661 | 0.649 | 0.554 | 0.554 | 0.601 | 0.608 | 0.614 | 0.474 | 0.515 | 0.533 | 0.505 | 0.549 |
| 100 | 0.510 | 0.502 | 0.505 | 0.492 | 0.496 | 0.644 | 0.622 | 0.650 | 0.661 | 0.637 | 0.557 | 0.566 | 0.589 | 0.626 | 0.608 | 0.493 | 0.539 | 0.548 | 0.520 | 0.549 |
| 110 | 0.499 | 0.501 | 0.502 | 0.506 | 0.494 | 0.637 | 0.613 | 0.641 | 0.652 | 0.634 | 0.567 | 0.584 | 0.589 | 0.603 | 0.603 | 0.492 | 0.534 | 0.542 | 0.526 | 0.545 |
| 120 | 0.509 | 0.515 | 0.494 | 0.510 | 0.519 | 0.639 | 0.628 | 0.646 | 0.649 | 0.629 | 0.567 | 0.584 | 0.599 | 0.597 | 0.597 | 0.509 | 0.521 | 0.547 | 0.527 | 0.538 |
| 130 | 0.497 | 0.495 | 0.502 | 0.508 | 0.527 | 0.631 | 0.623 | 0.639 | 0.652 | 0.673 | 0.559 | 0.587 | 0.592 | 0.599 | 0.597 | 0.516 | 0.516 | 0.551 | 0.545 | 0.550 |
| 140 | 0.489 | 0.496 | 0.503 | 0.521 | 0.525 | 0.636 | 0.626 | 0.633 | 0.648 | 0.667 | 0.573 | 0.591 | 0.582 | 0.581 | 0.592 | 0.52 | 0.531 | 0.560 | 0.540 | 0.540 |
| 150 | 0.504 | 0.502 | 0.498 | 0.504 | 0.509 | 0.640 | 0.636 | 0.628 | 0.654 | 0.667 | 0.573 | 0.591 | 0.589 | 0.576 | 0.592 | 0.534 | 0.549 | 0.539 | 0.532 | 0.533 |
| 160 | 0.497 | 0.494 | 0.508 | 0.516 | 0.524 | 0.621 | 0.621 | 0.633 | 0.646 | 0.667 | 0.557 | 0.582 | 0.596 | 0.586 | 0.588 | 0.536 | 0.545 | 0.551 | 0.541 | 0.537 |
| 170 | 0.506 | 0.511 | 0.505 | 0.512 | 0.525 | 0.624 | 0.617 | 0.626 | 0.642 | 0.673 | 0.555 | 0.577 | 0.585 | 0.593 | 0.583 | 0.524 | 0.525 | **0.566** | 0.559 | 0.539 |
| 180 | 0.512 | 0.532 | 0.507 | 0.527 | 0.528 | 0.630 | 0.622 | 0.623 | 0.642 | 0.658 | 0.574 | 0.590 | 0.593 | 0.593 | 0.597 | 0.554 | 0.555 | 0.556 | 0.521 | 0.533 |
| 190 | 0.504 | 0.511 | 0.509 | 0.529 | **0.539** | 0.634 | 0.617 | 0.646 | 0.643 | 0.670 | 0.566 | 0.584 | 0.608 | 0.599 | 0.606 | 0.55 | 0.563 | 0.548 | 0.522 | 0.529 |
| 200 | 0.513 | 0.527 | 0.505 | 0.524 | 0.534 | 0.637 | 0.619 | 0.647 | 0.645 | 0.675 | 0.555 | 0.591 | 0.606 | 0.605 | 0.612 | 0.524 | 0.545 | 0.551 | 0.517 | 0.528 |
|  | **A) Spectral bands (SP)**  **B) Spectral bands + Texture +Geo (SP + TXT + GEO)** | | | | | | **C) Deep learning features (FEA)**  **D) Deep learning features + Spectral bands (FEA +SP)** | | | | | | | **E) Deep learning features + Spectral bands + Texture + Geometry (FEA + SP +TXT +GEO)** | | | | | | |

Table A5. Percent improvement in Prediction scores of the environmental variables from pixel based to object based method (scale 60-180)

|  | Median grain size  (µm) | | | | Silt content  (% of volume <63µm) | | | | Biomass  (gADW/m^2^) | | | | Species richness  (no. of species/sample) | | | |
| --- | --- | --- | --- | --- | --- | --- | --- | --- | --- | --- | --- | --- | --- | --- | --- | --- |
| Scale | Spectral bands  (I) | Spectral bands + Features (II) | **(II-I)%** | II(Object) –  I (Pixel)  % | Spectral bands  (I) | Spectral bands + Features (II) | **(II-I)%** | II(Object) –  I (Pixel)  % | Spectral bands  (I) | Spectral bands + Features (II) | **(II-I)%** | II(Object) –  I((Pixel)  % | Spectral bands  (I) | Spectral bands + Features (II) | **(II-I)%** | II(Object) –  I (Pixel)  % |
| Pixel-based | | | | | | | | | | | | | | | | |
| - | 0.300 | 0.412 | 11.2 | - | 0.340 | 0.477 | 13.7 | - | 0.120 | 0.260 | 14.0 | - | 0.118 | 0.275 | 15.7 | - |
| Object-based | | | | | | | | | | | | | | | | |
| 60 | 0.434 | 0.488 | **5.4** | 18.8 | 0.393 | **0.509** | **11.6** | **16.9** | 0.241 | 0.305 | 6.4 | 18.5 | 0.259 | **0.358** | **9.9** | **24.0** |
| 70 | 0.449 | 0.488 | 3.9 | 18.8 | 0.399 | 0.508 | 10.9 | 16.8 | 0.233 | **0.311** | 7.8 | **19.1** | 0.270 | 0.346 | 7.6 | 22.8 |
| 80 | 0.476 | 0.497 | 2.1 | 19.7 | 0.392 | 0.487 | 9.5 | 14.7 | 0.248 | 0.293 | 4.5 | 17.3 | 0.281 | 0.341 | 6.0 | 22.3 |
| 90 | 0.496 | 0.495 | 0.1 | 19.5 | 0.392 | 0.482 | 9.0 | 14.2 | 0.219 | 0.280 | 6.1 | 12.0 | 0.279 | 0.329 | 5.0 | 21.1 |
| 100 | 0.497 | 0.502 | 0.5 | 20.2 | 0.394 | 0.477 | 8.3 | 13.7 | 0.216 | 0.261 | 4.5 | 14.1 | 0.278 | 0.309 | 3.1 | 19.1 |
| 120 | 0.489 | 0.499 | 1.0 | 19.9 | 0.385 | 0.469 | 8.4 | 12.9 | 0.184 | 0.261 | 7.7 | 14.1 | 0.266 | 0.297 | 3.1 | 17.9 |
| 140 | 0.497 | 0.518 | 2.1 | 21.8 | 0.411 | 0.482 | 7.1 | 14.2 | 0.187 | 0.252 | 6.5 | 13.2 | 0.281 | 0.301 | 2.0 | 18.3 |
| 160 | 0.505 | 0.516 | 1.1 | 21.6 | 0.414 | 0.489 | 7.5 | 14.9 | 0.135 | 0.223 | **8.8** | 10.3 | 0.249 | 0.271 | 2.2 | 15.3 |
| 180 | 0.509 | **0.535** | 2.6 | **23.5** | 0.448 | 0.489 | 4.1 | 14.9 | 0.141 | 0.208 | 6.7 | 8.8 | 0.246 | 0.255 | 0.9 | 13.7 |
